# Polysomnographic characteristics and treatment modalities in a referred population of children with trisomy 21

**DOI:** 10.3389/fped.2022.1109011

**Published:** 2023-01-10

**Authors:** Kaelyn Gaza, Jodi Gustave, Seema Rani, Abigail Strang, Aaron Chidekel

**Affiliations:** Division of Pulmonology and Sleep Medicine, Nemours Children's Hospital, Wilmington, DE, United States

**Keywords:** trisomy 21 (Down syndrome), osa, breathing abnormalities, cardiac, pediatrics - children

## Abstract

**Background:**

Children with Trisomy 21 (T21) are at an increased risk of sleep-disordered breathing (SDB), which can impact daily functioning and cause other health complications. Accordingly, it is imperative to diagnose and treat SDB in this population. Current guidelines recommend screening polysomnogram by age 4 or sooner if clinically indicated. There are limited published studies describing characteristics of SDB in children with T21, particularly in infants and young children.

**Objective:**

The objective of this study is to characterize SDB and treatment modalities in infants and young children with T21.

**Methods:**

This is a retrospective review of a cohort of children (≤60 months of age) with T21 who completed a polysomnogram (PSG) between 2015 and 2020 at a pediatric referral center. Demographic information, relevant medical history, polysomnography parameters, and treatment details of these children were collected from EMR. Descriptive and comparative statistics were calculated for the cohort; additional subgroup analysis was completed by age 0–35 months and 36–60 months.

**Results:**

Most of the cohort met criteria for sleep apnea (84.1%), and airway surgery was the most common treatment modality (71.4%). The mean AHI was high (21.4 events/hour) with a trend towards hypoventilation (mean EtCO_2_ = 55.9 mmHg; mean percentage of TST with EtCO2 > 50 mmHg 20.8%). Mean arousal index was elevated (32 events/hour). There were no significant differences in SDB by age when we compared children 0–35 months and 36–60 months.

**Conclusions:**

This cohort of referred children with T21 showed high prevalence of SDB with a trend towards hypoventilation and disrupted sleep quality with no significant differences by age. These data highlight the importance of maintaining a high index of suspicion for SDB in young patients with T21 and obtaining PSG testing to characterize sleep and breathing.

## Introduction

Sleep disordered breathing (SDB) ranges from primary snoring to obstructive sleep apnea (OSA) and may cause disruptions in sleep quality as well as gas exchange abnormalities. Multiple studies have reported high prevalence rates of SDB including Obstructive Sleep Apnea (OSA) in children with Trisomy 21 (T21) ranging from 43% to 96% (1–4). This prevalence is much higher compared with the general pediatric population (5, 6). There are several anatomical features and associated comorbidities in children with T21 which predispose this population to SDB. Multiple anatomic features including glossoptosis, enlarged lingual tonsils, midface hypoplasia, relative macroglossia and hypotonia are associated with SDB (7, 8). Children with T21 also are more likely to have co-morbidities including obesity, hypothyroidism, and gastrointestinal and cardiac co-morbidities, which infer increased risk for SDB (8–10).

Undiagnosed and untreated SDB is associated with consequent fatigue, neurocognitive issues, cardiac conditions, and failure to thrive. The neurocognitive sequelae include behavioral difficulties, problems with executive dysfunction, difficulty planning, poor memory, lowered verbal IQ, and limited cognitive flexibility (11–16). The cardiac consequences may include pulmonary hypertension and heart failure (16).

Current guidelines recommend screening polysomnogram (PSG) for children with T21 at the age of 4 years or earlier if there are clinical signs and symptoms (17). There are limited studies evaluating SDB in younger children (≤5 years old) with T21. Few studies done in younger children with T21 have found high rates of OSA; other studies report not only higher rates of prevalence of OSA but also a more severe degree of OSA in infants with T21 (1, 18).

The goal of this retrospective study is to describe clinical characteristics, PSG data, comorbid conditions, and treatment modalities in young children with Trisomy 21 (<60 months old) who had an initial baseline PSG performed at a single referral institution. Additional subgroup analysis separated the data by age group (0–35 months and 36–60 months).

## Methods

### Patient population

This IRB-approved, retrospective review examined children with T21 who were 60 months old or younger at the time of first PSG between 2015 and 2020 and included both symptomatic and asymptomatic patients. Eligible patients were identified *via* review of EMR (electronic medical record) with problem list containing ICD code for Trisomy 21 (Q90.0) at the time of the PSG and confirmed *via* manual chart review by a member of the research team.

### Data collection

Data collected for this study consisted of demographic information, relevant medical history, PSG data, and treatment information. All data were extracted retrospectively from the EMR *via* chart review by a member of the research team. PSG data was collected *via* sleep study report available in the EMR. All patients included in this study completed a clinical office visit with a pediatric sleep specialist prior to the PSG which includes clinical exam and documentation of relevant medical and surgical history. These visits are typically completed <2 months prior to the PSG. Clinical and demographic data were collected for this project *via* review of the problem list and documentation in the EMR at the time of the office visit.

The demographic information consisted of age, sex, race, ethnicity, height, height *z*-score, weight, weight *z*-score, BMI, BMI *z*-score, medications at the time of the PSG, and tobacco smoke exposure. *Z*-scores for height and weight were determined using Trisomy 21 growth charts while *z*-scores for BMI were determined using WHO growth charts.

Relevant medications included nasal steroids, antihistamines, montelukast, melatonin and other non-prescription sleep aids, inhaled medications, histamine-2 antagonists (hist-2), and proton pump inhibitors (PPI). These data were collected from the EMR as documented in the medication review at the clinical encounter immediately prior to the PSG. Relevant medical history included the existence of relevant pre-existing conditions, feeding tube prior to the first PSG, ENT visits, ENT surgeries prior to the first PSG, ENT surgery at any time, prematurity, pulmonary conditions, dysphagia and other gastrointestinal conditions, cardiac disease, thyroid conditions, and neurodevelopmental conditions. The comorbidities were defined based on review of the problem list in the EMR at the time of the visit as well as documented in the clinical notes at the time of the clinical visit immediately prior to the PSG. Prematurity was defined as being born prior to gestational age of 37 weeks. Pulmonary conditions consisted of (1) history of asthma or recurrent wheezing that was diagnosed by a physician or for which the patient was prescribed pulmonary inhaled medications, (2) bronchopulmonary dysplasia (BPD) or chronic lung disease of prematurity, and (3) structural airway abnormalities, specifically oropharyngeal malformations, laryngomalacia, laryngeal clefts, vocal cord paralysis, tracheomalacia, bronchomalacia, and tracheoesophageal fistula (TEF). Gastrointestinal conditions consisted of (1) reflux that was diagnosed by a physician or for which the patient was prescribed reflux medications, (2) gastrointestinal surgical repair, and (3) structural GI anomalies, specifically duodenal atresia, duodenal stenosis, TEF, intestinal malrotation, and Hirschsprung's disease. Cardiac conditions consisted of (1) pulmonary hypertension, (2) surgical repair of structural heart defects, and (3) congenital structural heart defects, specifically ASD, VSD, AV canal, TOF, coarctation of aorta, and PDA. PFOs were not included as congenital structural heart defects. Thyroid conditions consisted of hypothyroidism and hyperthyroidism. Neurodevelopmental conditions consisted of autism and ADHD. ENT surgery included myringotomy tube placement, adenoidectomy, tonsillectomy, adenotonsillectomy, and supraglottoplasty.

PSG was performed in an American Academy of Sleep Medicine-accredited sleep laboratory at Nemours Children's Hospital, Delaware. PSG is the gold standard for the diagnosis of OSA (17). Electroencephalography, electrooculography, electromyography, and electrocardiography were continuously recorded throughout the PSG. Respiratory effort was measured *via* respiratory inductance plethysmography and oxygen saturation was measured *via* a finger probe on a pulse oximeter. A nasal pressure cannula and a thermistor measured airflow, and snoring was measured *via* a microphone. The patients were monitored using by a polysomnographic technician during the entire duration of the study in a dark and comfortable environment. The raw data was reviewed and interpreted by a pediatric board-certified sleep medicine physician. Respiratory events were scored according to the American Academy of Sleep Medicine guidelines using pediatric scoring (19).

The PSG data collected included respiratory and sleep architecture data. The respiratory parameters consisted of the central apnea index (CAI), obstructive apnea index (OAI), hypopnea index (HI), apnea-hypopnea index (AHI), number of episodes of oxygen saturations less than 90% (# Episodes SpO_2_ < 90%), the percent of total sleep time spent below 90% saturation (TST with SpO_2_ < 90%), number of episodes of relative oxygen desaturations ≥3% (Episodes SpO_2_ desaturation ≥ 3%), SpO_2_ nadir, mean SpO_2_ in REM, mean SpO_2_ in NREM, peak End tidal CO_2_ (EtCO_2_) level, percent of total sleep time spent with a peak EtCO_2_ level above 50 torr (TST w/EtCO_2 _> 50 torr), periodic breathing time (PBT), percent of total sleep time spent with periodic breathing (TST with PBT), mean respiratory rate in REM (MRR REM), and mean respiratory rate in NREM (MRR NREM), periodic limb movements of sleep index (PLMI), periodic limb movements associated with arousals (PLM assoc. with arousals) for children above the age 2 years. Sleep architecture included the patient's total sleep time (TST), sleep efficiency (SE), sleep latency, percentage of TST in REM, percentage of TST in N3, and arousal index. Sleep efficiency was considered decreased if it was <90%. Severe sleep apnea was noted if the AHI was ≥10/hour. Potential treatment options were supplemental oxygen, airway surgery, positive airway pressure (PAP), intranasal corticosteroids, and other. Airway surgery consisted of adenoidectomy alone, adenotonsillectomy, and supraglottoplasty.

### Analysis

All analysis was performed using group-generated R scripts. Descriptive statistics consisted of mean, median, standard deviation, and percentages where appropriate. In subgroup analysis, the younger (0–35 months) patients were compared to the older (36–60 months) patients using 2-sample Wilcoxon-rank sum test after tests for normality were performed using the Shapiro-Wilk test. A *p* value of ≤0.05 was considered statistically significant.

## Results

68 children were found to fit all inclusion criteria; four were excluded due to incomplete PSG data. Therefore, the cohort analyzed consisted of a baseline PSG in 64 children with T21.

### Demographic and clinical data

The full cohort was predominantly female (54.7%), white (61.9%), and non-Hispanic or Latino (71.9%). 14.3% of the cohort were black, and 22.2% were listed as “other.” The mean age of the participants was 30.8 months (median = 30.5, SD = 16.4). See Table 1 for descriptive statistics for height, weight, and BMI and *z*-scores for those parameters. 63.5% were taking relevant medications with the most common being inhaled respiratory therapies (39.7%). Roughly a quarter had a history of tobacco smoke exposure.

**Table 1:** Anthropometric information for total cohort (*n* = 64).

	Mean	Median	SD
Height	0.819 m	0.832 m	0.126 m
Height *Z*-Score	−0.084	−0.02	1.06
Weight	12 kg	12.4 kg	3.73 kg
Weight *Z*-Score	0.194	0.25	0.823
BMI	17.4 kg/m^2	17.2 kg/m^2	1.78 kg/m^2
BMI *Z*-Score	0.973	0.985	1.20

### Comorbid conditions

The most prevalent comorbidities include congenital heart disease (78.1%) and asthma/wheezing history (43.8%). Table 2 details the percentage of patients with each comorbid condition.

**Table 2:** Comorbid conditions (%).

Pulmonary Conditions			
Overall	Structural Abnormalities	Asthma	Chronic Lung Disease
53.12	20.3	43.8	10.9
Gastrointestinal Conditions			
Overall	Reflux	Structural Abnormalities	Surgery
65.6	65.6	3.1	6.3
Congenital Heart Disease			
Overall	Hypertension	Structural Abnormalities	Surgery
79.7	14.1	78.1	28.1
Thyroid Conditions			
Overall	Hypothyroidism	Hyperthyroidism	
7.81	7.81	0	
Miscellaneous			
Prematurity	Dysphagia		
26.6	34.38		

### Relevant medical and surgical history

In this cohort, many children were referred for PSG with history of airway concerns including enlarged tonsils (62.5%), snoring (81.2%), and restless sleep (59.4%). 18.8% were considered to be asymptomatic and referred due to age and history of Trisomy 21. Noisy breathing was present in approximately 25% of the population. Asthma or wheezing was present in 43.8% of patients of which most (89.3%) were treated with inhaled medications. Reflux was diagnosed in 64.6%, and 42.9% were treated with medical therapy. Roughly half of the patients had a feeding tube (either nasogastric tube or surgically-placed tube) at some point prior to the PSG.

### PSG respiratory parameters

Table 3 contains respiratory parameters of the PSG. In general, these patients had high rates of obstructive apnea and hypopnea events (mean = 21.4, median = 17.8, SD = 19.1). The hypopnea index is high (HI: mean = 15.2, median = 12, SD = 16.4). The SpO_2_ nadir was moderately reduced with a mean value of 86.2% (median = 88.5%, SD = 7.8%); however most had saturations above 90% for majority of the study (Duration of SpO2 below 90%; mean = 1.96% of TST, median = 0% of TST, SD = 7.33% of TST). Mean oxygen saturation recorded was 97% (median = 97%, SD = 2.1% in REM; 1.7% in NREM). In addition to experiencing frequent apnea and hypopnea episodes, the patients also demonstrated borderline abnormal ventilation an elevated mean peak EtCO2 (mean = 55.9 torr, median = 54.8 torr, SD = 6.75 torr) and percentage of time above 50 torr (mean = 20.8%, median = 5.6%, SD = 29.4%).

**Table 3:** Polysomnography parameters.

PSG Parameter	All patients (0–60 months) Mean/Median (SD)	Younger Patients (0–35 months) Mean/Median (SD)	Older Patients (36–60 months) Mean/Median (SD)	*p*-value
Total Sleep Time (min)	401/413 (64.6)	395/406 (67.7)	409/419 (60.9)	0.29
Sleep Efficiency (%)	83.4/84.2 (9.08)	82.4/83.7 (9.07)	84.6/86.5 (9.12)	0.31
CAI	2.89/2.01 (3.07)	2.22/2 (1.54)	3.7/2.02 (4.13)	0.45
OAI	3.27/1.08 (5.59)	4.1/1.52 (6.86)	2.27/0.86 (3.36)	0.15
HI	15.2/12 (16.4)	17.8/12 (20.2)	12.1/11.9 (9.59)	0.51
AHI	21.4/17.8 (19.1)	24.2/18 (23.2)	18.1/17.7 (12)	0.62
Episodes SpO_2_ < 90% (%)	**26.6/1.5** **(72.2)**	**42.2/3** **(94.7)**	**7.79/0** **(13.2)**	**0**.**05**
TST with SpO_2_ < 90% (%)	1.96/0 (7.33)	3.37/0.03(9.72)	0.26/0(0.80)	0.13
Episodes SpO_2_ Desat. >/= 3%	94.5/63.5 (106)	114/61(131)	71.3/66(57.4)	0.54
SpO_2_ Nadir (%)	86.2/88.5 (7.79)	84.5/87 (9.22)	88.2/90 (5.07)	0.12
Mean SpO_2_ REM (%)	96.7/97 (2.07)	96.4/97 (2.3)	97.1/97.5 (1.72)	0.18
Mean SpO_2_ NREM (%)	96.7/97 (1.7)	96.6/97(2)	96.9/97 (1.26)	0.87
Peak EtCO_2_ (torr)	55.9/54.8 (6.75)	55.5/55.5 (5.3)	56.3/54.5 (8.18)	0.73
TST EtCO_2 _> 50 torr (%)	20.8/5.6 (29.4)	19.7/4.02 (30.5)	22.1/9.56 (28.6)	0.64
Sleep Latency (min)	21.4/9.25 (26.5)	14.2/2.7 (20.1)	30/24.5 (30.8)	0.05
TST in REM (%)	14.4/13.6 (6.43)	15.5/15.3 (6.34)	13.2/13.1 (6.43)	0.25
TST in N3 (%)	42.7/41.1 (15.1)	39.5/37.7 (14)	46.5/43.7 (15.8)	0.07
Arousal Index	32/29.2 (17.7)	34/30.1 (20.8)	29.7/28.3 (13.1)	0.58
Mean RR REM	**18.8/16.6** **(5.9)**	**21.3/19.2** **(6.8)**	**15.6/15.6** **(1.69)**	**<0**.**01**
Mean RR NREM	**19.1/17** **(6.27)**	**20.8/18.9** **(6.72)**	**17/16** **(5.04)**	**<0**.**01**

### PSG sleep architecture

Table 3 includes all descriptive statistics for the cohort's sleep architecture. The majority (94%) had TST >300 min. 78.1% of studies showed decreased sleep efficiency of <90%. The sleep latency (mean = 21.4 min, median = 9.25 min, SD = 26.5 min) was within normal range, but the arousal index (mean = 32, median = 29.2, SD = 17.7) was elevated (20). The patients spent more time in N3 (mean = 42.7%, median = 41.1%, SD 15.1%) and less time in REM (mean = 14.4%, median = 13.6%, SD = 6.4%) than expected for the mean age (20).

### Treatment modalities

A majority of the cohort was found to have OSA (84.1%), and 67.2% had severe sleep apnea (defined as AHI >10 events/hour). Airway surgery was the most common treatment choice (65.1%) followed by intranasal corticosteroids (11.1%), PAP therapy (6.4%), other (4.8%), and supplemental oxygen (3.2%). Many patients (12.5%) received more than one treatment modality. Approximately 30% of patients were followed with clinical observation after the baseline PSG.

### Subgroup analysis by age

The entire cohort was sub-divided into younger (0–35 months of age) and older (36–60 months of age) cohorts with 35 and 29 patients, respectively. There were no significant differences in the majority of PSG parameters and specifically no differences in rates of OSA. See Table 3 for full comparison of all PSG parameters between the two groups. One notable difference between the two group's PSG parameters was in the number of desaturations less than 90% with the younger cohort having a mean of 42.2 compared to the older group having a mean of 7.8 (*p* = 0.05). As expected physiologically, the mean respiratory rates were higher in younger cohort in both REM (21.3 vs. 15.6; *p* < 0.01) and NREM (20.8 vs. 17; *p* < 0.01) Another important difference between the two cohorts was the diversity of treatment modalities. The older cohort almost exclusively underwent an AT or clinical observation with only 3.45% of patients receiving an alternative strategy. In comparison, 28.6% of the younger cohort received treatment other than an AT or clinical observation (including supplemental oxygen, CPAP).

## Discussion

In this cohort of children <60 months with T21, a majority (84%) demonstrated OSA on baseline PSG. Of these, 67.2% met criteria for severe OSA, which is similar to prior studies showing high rates of SDB in this population (2, 4, 9, 21). The high rate of OSA was not age-dependent with high rates described in both the 0–35 month and 36–60 month subgroups.

Polysomnogram data in infants and younger children with T21 have not been well studied; however studies published in these young age groups show high prevalence of OSA (21, 22). In this study, not only were there high rates of OSA in the younger group, but also high rates of increased severity of OSA with a mean AHI of 24.2. The combined cohort had borderline abnormal gas exchange with a trend toward hypoventilation (peak EtCO_2_: mean = 55.9 torr, median = 54.8 torr, SD = 6.75 torr; TST with EtCO_2 _> 50 torr: mean = 20.8%, median = 5.6%, SD = 29.4%).

Sleep disturbances were also prevalent in this cohort. Similar to prior studies, this cohort demonstrated disrupted sleep with high mean arousal index and abnormal sleep architecture with increased duration of N3 sleep and reduced duration of REM. The mean sleep efficiency of the cohort was lower than expected. Testing procedures and other behavioral comorbidities may be potential underlying factors contributing to this finding. Notably, most children (94%) were able to sleep for >300 min.

The higher rates of OSA may be attributed to a medically-complex referred population analyzed in our cohort. There were high rates of comorbidities with nearly all patients (95.3%) having one comorbid condition. Overall, congenital heart defects were the most common comorbidity (78.1%), but reflux (65.62%) and asthma/wheezing (43.8%) were also very prevalent in this cohort, both which may predispose to SDB. Compared to other published studies, this cohort includes large number of infants and children with T21 who are surgically naïve (with the exception of one child who had a prior supraglottoplasty). In addition, this study data adds to further characterize various comorbidities and sleep disordered breathing as well as detailing sleep architecture in younger children and infants with T21.

Overall, the prevalence of OSA was high in this cohort of referred children with T21, including younger children and infants aged 0–35 months. These results call for continued diligent screening for sleep disordered breathing children with T21 beginning in infancy and maintaining high level of clinical suspicion for sleep apnea in infants and younger toddlers, as it is known that parental report of sleep symptoms tend to underestimate PSG results. Also, given the high prevalence of associated Pulmonary, GI and Cardiac conditions in this cohort, there should be a high index of suspicion of SDB in patients with these comorbidities. Further studies are warranted to determine which comorbidities may cause highest risk of SDB in this unique population.

## Data Availability

The original contributions presented in the study are included in the article/Supplementary Material, further inquiries can be directed to the corresponding author/s.
